# Genistein Ameliorates Renal Fibrosis Through Regulation Snail via m6A RNA Demethylase ALKBH5

**DOI:** 10.3389/fphar.2020.579265

**Published:** 2020-11-19

**Authors:** Yichun Ning, Jing Chen, Yiqin Shi, Nana Song, Xiaofang Yu, Yi Fang, Xiaoqiang Ding

**Affiliations:** ^1^ Department of Nephrology, Zhongshan Hospital, Fudan University, Shanghai, China; ^2^ Shanghai Medical Center of Kidney, Shanghai, China; ^3^ Shanghai Institute of Kidney and Dialysis, Shanghai, China; ^4^ Shanghai Key Laboratory of Kidney and Blood Purification, Shanghai, China; ^5^ Hemodialysis Quality Control Center of Shanghai, Shanghai, China

**Keywords:** renal fibrosis, genistein, RNA methylation, epithelial-to-mesenchymal transition, ALKBH5

## Abstract

Renal tubule-interstitial fibrosis is related to chronic kidney disease progression and a typical feature of the aging kidney. Epigenetic modifications of fibrosis-prone genes regulate the development of renal fibrosis. As a kind of “epigenetic diet”, soy isoflavone genistein was reported to have renal protective action and epigenetic-modulating effects. However, its renal protection role and underlying mechanisms are yet to be fully clarified. Herein, we showed that genistein exhibits a demonstrable anti-fibrotic effect on kidney *in vivo* UUO (unilateral ureteral occlusion) model and renal epithelial cells *in vitro* model. The mechanism is strongly associated with epithelial-to-mesenchymal transition and m6A RNA demethylase ALKBH5. Mouse fibrotic kidneys induced by UUO exhibited adverse expression of renal fibrosis-related proteins and significant increases in the total m6A level. As an eraser, ALKBH5 showed severer suppression in the renal fibrosis process. However, genistein pretreatment restored ALKBH5 loss remarkably and reduced renal fibrosis, abnormal protein, and inflammatory markers. The examination of possible mechanisms revealed that genistein promoted ALKBH5 and maybe induced the level of mRNA m6A methylation in some epithelial-to-mesenchymal transition-related transcription factors. We found snail was the critical regulator and critical for the protective role of genistein. To verify the relationship between ALKBH5 and snail, we generated knockdown and overexpression of ALKBH5 cells *in vitro*. ALKBH5 knockdown enhanced the mesenchymal phenotype marker α-smooth muscle actin and snail expression. In agreement, overexpression ALKBH5 increased epithelial adhesion molecule E-cadherin and reduced snail expression. In conclusion, genistein increased renal ALKBH5 expression in UUO-induced renal fibrosis and reduced RNA m6A levels and ameliorates renal damages.

## Introduction

Renal tubule-interstitial fibrosis is strongly related to the progression of chronic kidney disease (CKD) and is a typical character of renal aging (Humphreys, 2018). Kidneys with ureteral obstruction progress developing a tubule-interstitial injury. As a well-characterized animal model of renal injury, unilateral ureteral obstruction (UUO) is commonly used to examine the pathological processes of tubule-interstitial fibrosis (Klahr and Morrissey, 2002; Bascands and Schanstra, 2005). Obstructed renal tissue damage is accompanied by tubular degeneration and progressive interstitial fibrosis. And this is characterized by excessive production and deposition of extracellular matrix (ECM) in the interstitium (Humphreys, 2018). Proteins of ECM, including collagens and fibronectin, are produced by myofibroblasts. Previous studies have shown that these myofibroblasts stem from transformed epithelial cells or resident interstitial fibroblasts, which is known as the epithelial-to-mesenchymal transition (EMT) (Kalluri and Neilson, 2003). The pathological process in UUO renal damage is associated with EMT, in which there is an increase of α-smooth muscle actin (α-SMA) expression and down-expression in E-cadherin (Strutz et al., 2002). As a result of a loss in epithelial-cell characteristics, myofibroblasts’ proliferative and migration ability increase resulting in the production and deposition of several ECM in the renal interstitium. Meanwhile, many immune cells infiltrate and secret numerous profibrotic factors (Chevalier, 2006).

Genistein (4,5,7-trihydroxyisoflavone), a soy isoflavone has been extensively studied. It is a polyphenolic non-steroidal compound that is widely used as a dietary supplement and even been called an “epigenetic diet” (Ganai and Farooqi, 2015; Li et al., 2019). Studies show that genistein can be used for the prevention of metabolic disorders associated with cancer (Pavese et al., 2010), cardiovascular disease (CVD) (Gencel et al., 2012), obesity (Orgaard and Jensen, 2008), and diabetes (Stephenson et al., 2005; Rehman et al., 2019). Also, it protects against acute kidney injury (AKI) such as ischemia/reperfusion (I/R)-induced renal injury, cisplatin, and radiation-induced nephrotoxicity (Sung et al., 2008; Canyilmaz et al., 2016; Li et al., 2017). A recent study demonstrated that genistein could protect against UUO-induced renal fibrosis by modulating the expression of klotho via epigenetic processes involving DNA methylation and histone modifications (Li et al., 2019). Genistein has many advantageous characteristics; it is low in toxicity and is widely available, which justifies its great potential in clinical application. However, the precise function and underlying mechanism of genistein in CKD has not been fully elucidated.

N6-methyladenosine (m6A) is by far the most ubiquitous post-transcriptional methylation of mRNA in eukaryotic cells (Cao et al., 2016). m6A-dependent mRNA modification is a critical process particularly in mammals. It modulates several biological processes, such as self-renewal and differentiation, DNA damage response, tissue development, RNA–protein interactions, and primary microRNA processing by regulating RNA splicing, stability, translocation, and translation into protein (Cao et al., 2016). The modification of m6A is reversible and is regulated by the “writers” (methyltransferases), “readers” (m6A-binding proteins) and “erasers” (demethylases), which are thought to be vital in the development of various diseases, such as cancer (Liu et al., 2018), CVD (Dorn et al., 2019) and aging (Min et al., 2018). Recent evidence shows that m6A is linked to AKI (Wang et al., 2019; Xu et al., 2020). ALKBH5, one of m6A demethylases, has been reported in multiple tumors and plays a crucial role in EMT regulation via the suppression of various epithelial markers (Lin et al., 2019; Tang et al., 2020).

In this study, we assessed how genistein mitigates renal interstitial fibrosis and the mechanism underlying epigenetic regulation. We found m6A eraser ALKBH5 low expression in the obstructed kidneys; however, genistein can make ALKBH5 recovery. Considering that the EMT process participates in renal interstitial fibrosis development, which leads to renal failure, treatment strategies aiming at its suppression could help in the prevention and management of the disease. The findings of the present study show that genistein can be effective in the treatment and prevention of renal failure linked to fibrosis.

## Materials and Methods

### Animals

Approval for the execution of the current study was provided by the Animal Care and Use Committee of Fudan University, Shanghai, China. It was conducted based on the NIH Guide for the Care and Use of Laboratory Animals. Male mice (C57BL/6; age, 8 weeks; weight, 20–25 g) were obtained from the Animal Center of Fudan University. The mice were housed in an aseptic facility with controlled humidity, temperature, and 12-h light/dark cycle. And they were allowed free access to food and water. Twenty-four mice were randomly separated into four groups and six mice in each group: sham-operated (sham), sham + Genistein (Gen, 10 mg/kg), UUO, and UUO + Gen (10 mg/kg). Genistein (Sigma Aldrich, St. Louis, MO, USA) was dissolved in 0.9% sodium chloride supplemented with 1% dimethylsulphoxide and administered via intraperitoneal injection (i.p.) 24 h prior to the UUO and this continued for seven days. Mice were anesthetized with 0.1% sodium pentobarbital to minimize the pain. We made a left flank incision of the mice in the UUO/UUO + Gen group to expose the left ureter. Next, the ureter was completely ligated using silk sutures (double 5–0) at the ureteropelvic junction. After laying the bowel back in place, the skin and muscles were closed with sterile surgical of 3–0 nylon. The sham-operated mice were also subjected to the same procedure except that their ureters were not ligated. The kidney and blood samples were harvested at seventh-day post-UUO or sham surgery.

### Cell Culture, Treatment, and Transfection

Human kidney tubular HK2 cells (ATCC, USA) were grown in DMEM/F12 containing 10% FBS (fetal bovine serum) and 1% penicillin/streptomycin (Gibco, USA). The culture was incubated at 37°C in 5% CO_2_. The cells were treated with genistein (15 μM) for 24 h with or without TGF-β (BD Biosciences, USA, 5 ng/ml). Next, the cells were transfected with the synthesized human ALKBH5 siRNA oligonucleotides and the scrambled oligonucleotides at final concentrations of 50 nM using Lipofectamine 3,000 as per the methods described by the manufacturer. To generate ALKBH5 overexpression cells, HK2 cells were transfected with pcDNA/ALKBH5 or vector control through the liposome-mediated transfection. Transfected cells were subjected to drug treatments or not after 48 h of transfection. Finally, ALKBH5 expression was checked by conducting a western blot assay.

### Assessment of Renal Function

The concentrations of blood urea nitrogen (BUN) and Serum creatinine (Cre) were measured using the QuantiChromTM Urea/Creatinine Assay Kit (BioAssay Systems, USA) as per the methods described by the manufacturer.

### Histological Examination

Samples of renal tissue were prepared primarily as before. For light microscopic observation, sections (4 μm) were stained with H&E (hematoxylin and eosin) and Masson’s trichrome. The images were interpreted after being studied under a light microscope by two independent and experienced renal pathologists who were blinded to the study protocol. For semiquantitative analysis of the frequency and severity of the renal injury, 10 high-magnification (×200) non-overlapping fields of the cortex and the outer stripe of the outer medulla were randomly selected for each animal and analyzed by ImageJ software (NIH). Degree of renal injury was graded on a scale from 0 to 4: 0 = normal, 1 ≤ 25% of the cortex, 2 = >25%–50% of the cortex, 3 = ≥50% to <75% of the cortex, 4 ≥ 75% of the renal cortex (Schwalm et al., 2017). Renal fibrosis was calculated as the ratio of collagen deposition (blue color area in Masson’s trichrome-stained sections) over the whole field area based on ten randomly selected fields, thereafter; the mean scores from five sections per animal were calculated (Wongmekiat et al., 2013).

### Immunohistochemistry Staining

The IHC was performed according to our previous study (Ning et al., 2018). We incubated the slides with an optimal dilution primary antibody against fibronectin (Abcam, United Kingdom, 1:100).

### Western Blot Assay

This assay was conducted based on the methods described previously (Ning et al., 2018). Primary antibodies used in the present study included anti-E-cadherin, pSmad2/3, and TGF-β (Cell Signaling Technology, USA, 1:1,000), α-SMA, ALKBH5, snail, and GAPDH (Abcam, United Kingdom, 1:1,000).

### RNA Isolation and Quantitative Real-Time RT-PCR

We used Trizol (Sigma Aldrich, St. Louis, MO, USA) for the isolation of total RNA. Next, RT-PCR was conducted as described previously (Ning et al., 2018). Finally, 2(–ΔΔCT) method was used to determine relative mRNA levels. Primer sequences used at this stage are displayed in Table 1.

**TABLE 1:** Primer sequences used in this study.

Gene	Forward primer	Reverse primer
Collagen I	5′-TGTTGGCCCATCTGGTAAAGA-3′	5′-CAGGGAATCCGATGTTGCC-3′
IL-1β	5′-ACTACAGGCTCCGAGATGAA-3′	5′-TGGGTCCGACAGCACGAGGC-3′
MCP-1	5′-TCCCAATGAGTAGGCTGGAG-3′	5′-AGTGCTTGAGGTGGTTGTGG-3′
TNF-α	5′-CCTGGCCAACGGCATGGATC-3′	5′-CGGCTGGCACCACTCGTTGG-3′
Alkbh5	5′-GTCCCGGGACAACTACAAGG-3′	5′-GATGTGGATGGGGTCAACGT-3′
GAPDH	5′-TGCACCACCAACTGCTTAG-3′	5′-GGATGCAGGGATGATGTTC-3′

### Measurement of m6A Modification

UPLC-MS/MS analysis of m6A was performed as previously described (Chen et al., 2019).

### Data Analysis

Statistical analyses were performed using SPSS software version 16.0. Results from three independent experiments were averaged and presented as mean ± SD (standard deviation). Differences within and between groups were analyzed using ANOVA (two-way analysis of variance) and Student’s t-test, respectively. A *p*-value of less than 0.05 indicated statistical significance.

## Results

### Genistein Ameliorated the Physiological and Pathological Lesions After Unilateral Ureteral Occlusion

**FIGURE 1:**
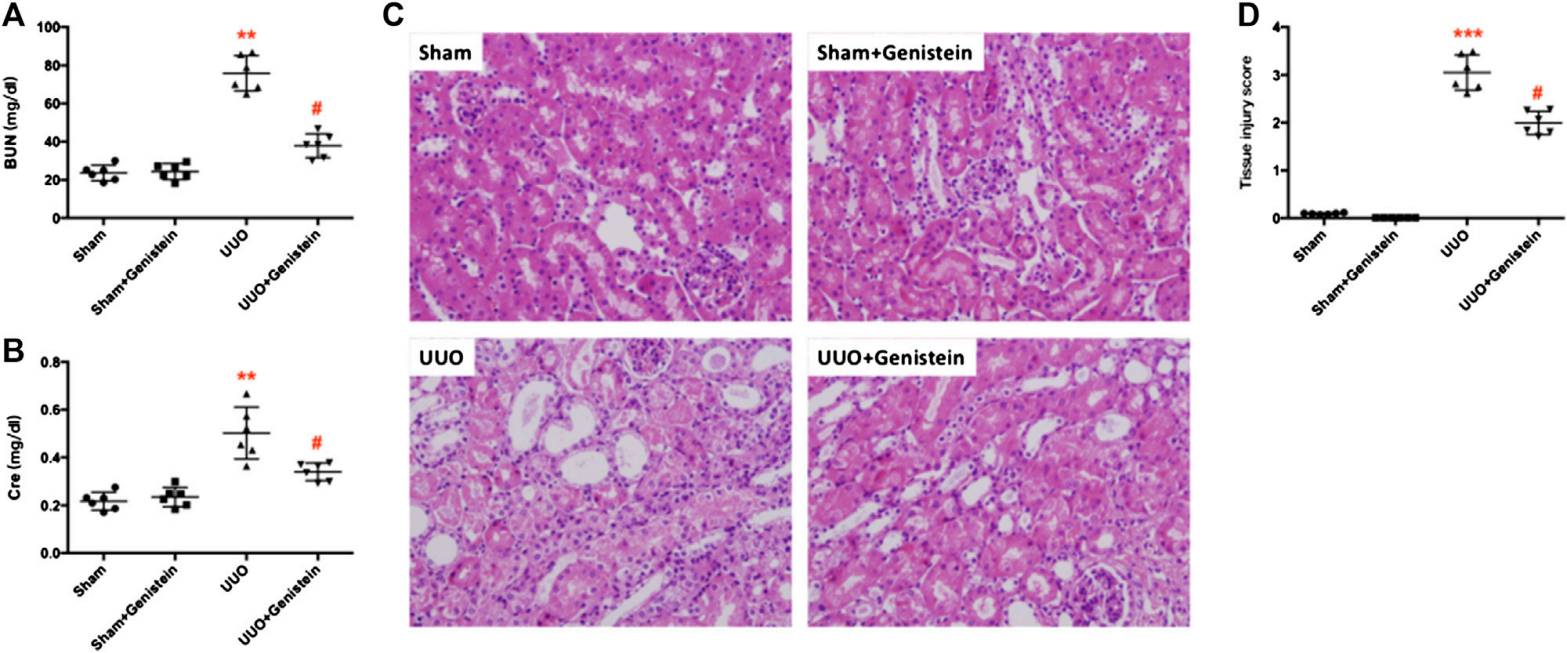
Genistein ameliorated the physiological and pathological lesions after UUO. **(A)** Average concentrations of blood urine nitrogen (BUN) and **(B)** serum creatinine (Cre) from four groups of experimental mice. **(C)** Representative HE-stained renal sections (200×) **(D)** Quantification of mouse kidney injury score. Data are presented as mean ± SD (n = 6, *p < 0.05, **p < 0.01 vs. sham; #*p* < 0.05 vs. UUO group).

Functional and histopathologic examinations were conducted to verify the protective effect of genistein. Increased blood urine nitrogen (BUN) and serum creatinine indicated the physiological lesions flowing UUO; however, the pretreatment of genistein significantly improved renal dysfunction (Figures 1A,B). The H&E staining indicated a normal renal cortex in the sham and sham + Gen group. Renal tubular damage occurred in the obstructed kidney, which included atrophy, tubular dilatation, epithelial cell desquamation, and deposition of hyaline in the tubular lumen (Figure 1C). Genistein pretreatment significantly ameliorated the above pathological lesions in the UUO + Gen group relative to the UUO group (Figures 1C,D; tissue injury score from 3.05 ± 0.15 to 1.99 ± 0.10; *p* < 0.05).

### Genistein Suppresses Interstitial Fibrosis with Unilateral Ureteral Occlusion

**FIGURE 2:**
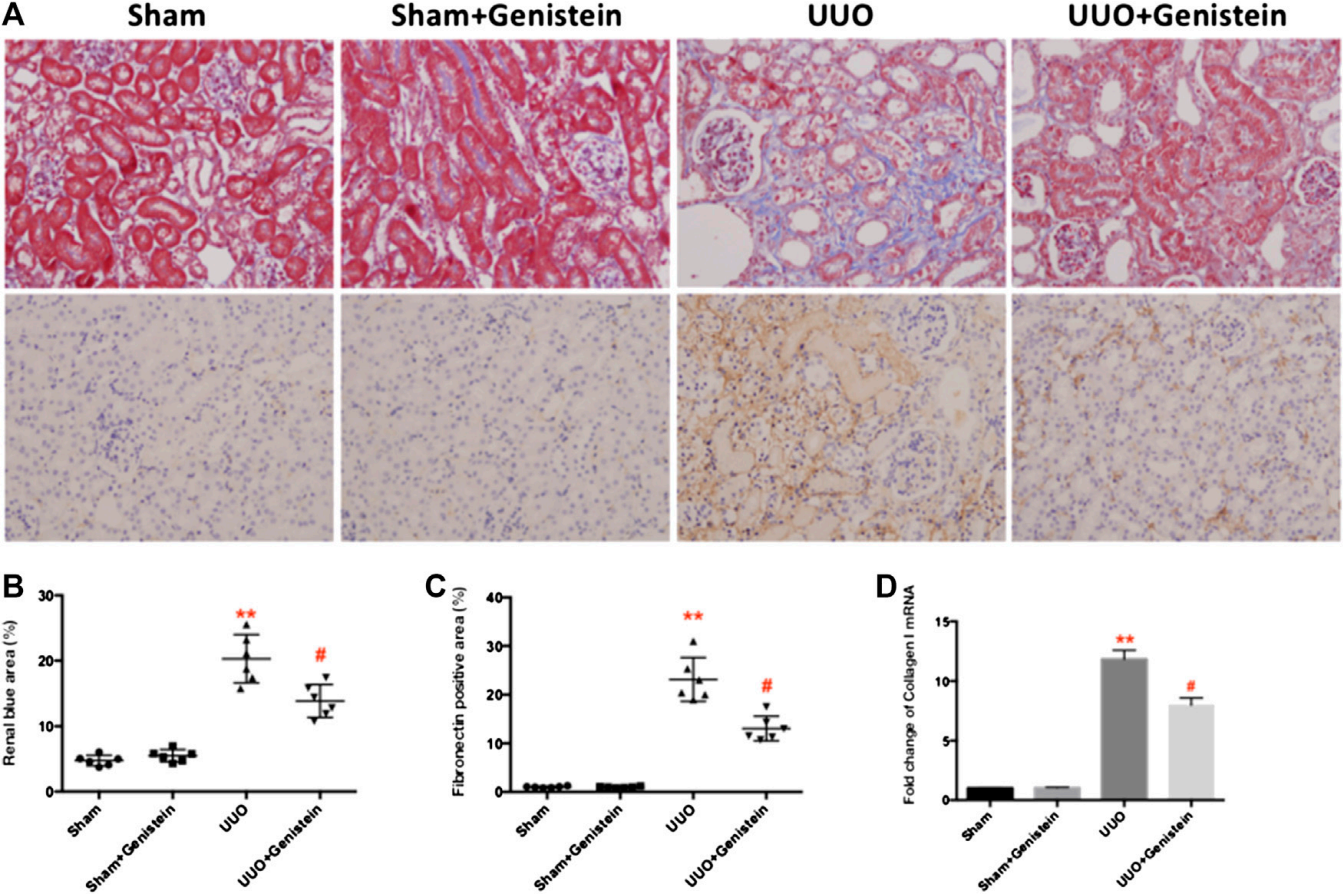
Genistein suppresses interstitial fibrosis. **(A)** Masson’s trichrome-stained and IHC of fibronectin in kidney sections (200×). Interstitial fibrosis lesions stained in blue and brown color are indicated respectively. **(B)** Quantifications of blue color area (%). **(C)** Quantifications of fibronectin positive area (%). **(D)** Renal expressions of collagen I mRNA from the experimental mice as revealed by RT-PCR with GAPDH. Data are presented as mean ± SD (n = 6, *p < 0.05, **p < 0.01 vs. sham; #*p* < 0.05 vs. UUO group).

Fibrosis examination of renal tissues was made by using Masson’s trichrome staining. The UUO group exhibited prominent tubule-interstitial fibrosis, and this was observed as an intense deposition of collagen in the interstitium (Figure 2A). Furthermore, analysis of semiquantitative verified that mice in the UUO-only group developed severe tubule-interstitial fibrosis, while only mild renal fibrosis in UUO + Gen group was detected on the seventh day after surgery (Figure 2B; the percent of the blue area from 20.3 ± 1.5 to 13.8 ± 1.0; *p* < 0.05). Subsequently, fibronectin (an ECM protein) expression was assessed by IHC in the renal tissues. A strong positive was observed in the interstitium bordering the dilated tubules. Expectedly, staining in the UUO group was more intense compared to the UUO + Gen group (Figures 2A,C). Consistent with the results of the mRNA levels of collagen I was increased in UUO mice (Figure 2D).

### Genistein Decreases Epithelial-to-Mesenchymal Transition and Reduced Inflammation by Unilateral Ureteral Occlusion

**FIGURE 3:**
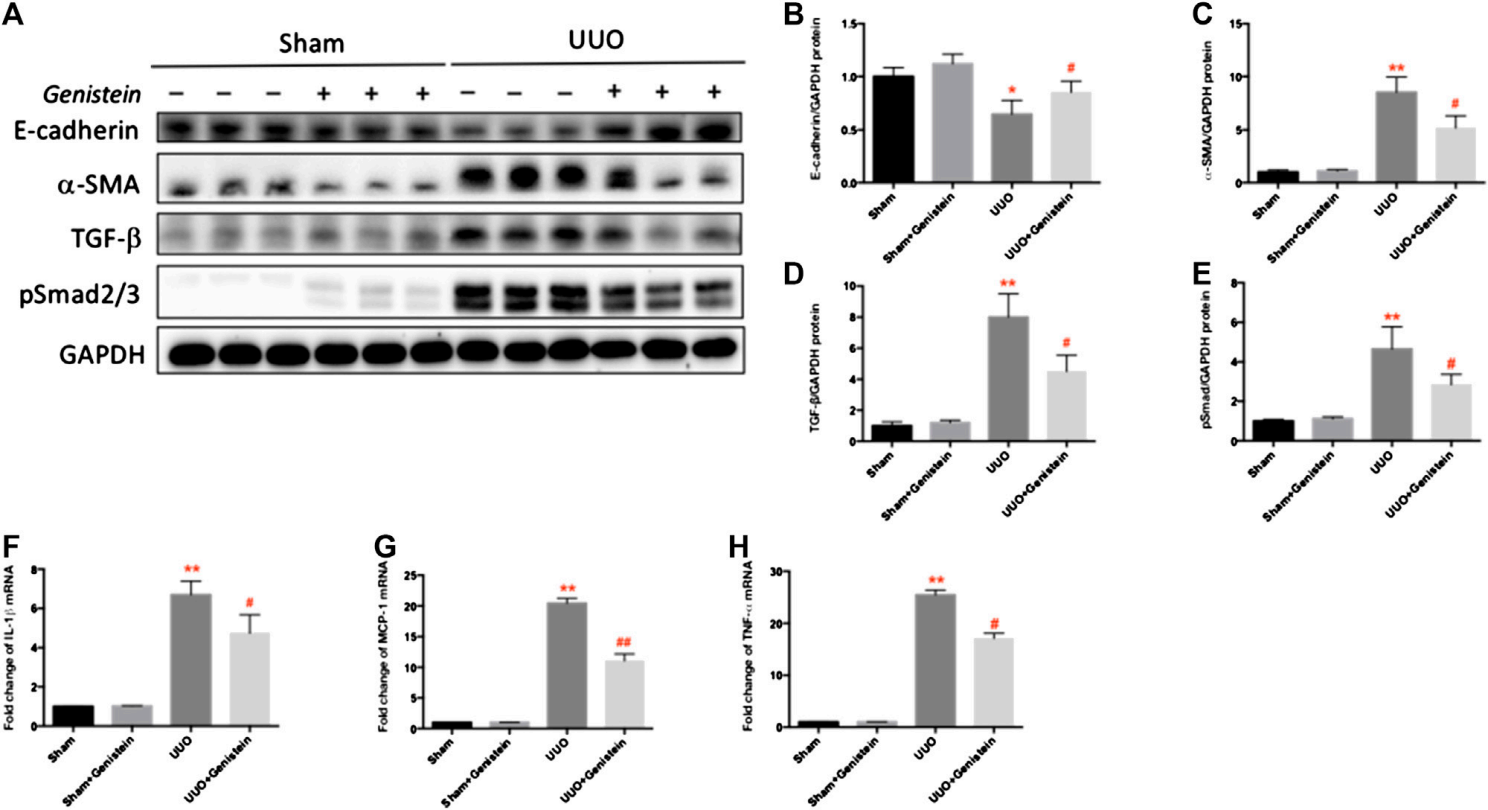
Genistein decreases EMT and reduced inflammation by UUO. **(A)** The protein expressions of E-cadherin, α-SMA, TGF-β, and pSmad2/3 as revealed by Western blot in renal tissue. The protein expressions of E-cadherin **(B)**, α-SMA **(C)**, TGF-β **(D)** pSmad2/3 **(E)** were quantitated by ImageJ software. The mRNA expression of IL-1β **(F)**, MCP-1 **(G)**, and TNF-α **(H)** in renal tissue were detected by RT-PCR. Data are presented as mean ± SD (n = 6, *p < 0.05, **p < 0.01 vs. sham; #*p* < 0.05, ##*p* < 0.01 vs. UUO group).

EMT is characterized by a loss of epithelial marker (i.e., E-cadherin) and overexpression of the mesenchymal marker (i.e., α-SMA). We made the analyses of total kidney lysates using western blot and found that E-cadherin expression was suppressed while α-SMA expression was enhanced in the UUO group; by contrast, in UUO + Gen mice, these effects were moderately ameliorated by Genistein (Figures 3A–C– C). TGF-β, a potent profibrotic cytokine, facilitates EMT by modulating the ECM protein expression directly in kidneys after UUO. Western blot analysis revealed a marked TGF-β induction in the UUO group; however, only a slight response was observed in the UUO + Gen group (Figures 3A,D). Given that the Smad signaling module is a pivotal modulator of fibrotic TGF-β signaling, we further analyzed the expression and subsequent Smad protein activation. Western blot analysis of total kidney extracts indicated that UUO led to a considerably high expression of pSmad2/3 in the obstructed kidneys; however, genistein treatment relatively restored these effects (Figures 3A,E). We detected the expression of proinflammatory cytokines, such as MCP-1, TNF-α, and IL-1β via RT-PCR to confirm whether genistein could attenuate UUO-induced inflammation. The mRNA levels of MCP-1, TNF-α, and IL-1β in the UUO group were higher compared to the sham group (Figures 3F–H). The mRNA level of IL-10, an anti-inflammation marker, was increased markedly in the kidney after UUO. However, genistein had no effect on IL-10 formation (not shown). Genistein treatment partially ameliorated these inflammatory factors secretion.

### Genistein Is Involved in m6A Modification and Increased m6A ALKBH5 Following Unilateral Ureteral Occlusion

**FIGURE 4:**
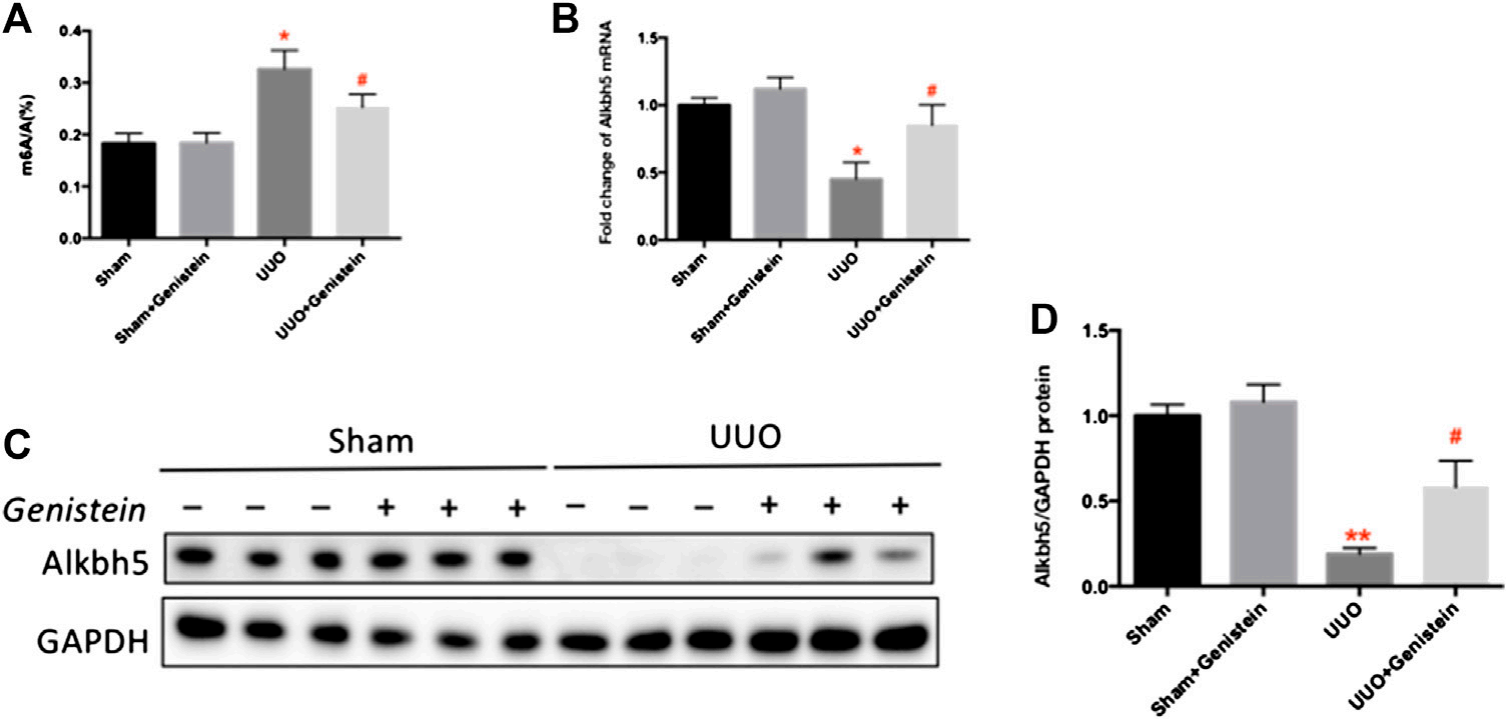
Genistein is involved in m6A modification and increased ALKBH5 following UUO. **(A)** The quantification of m6A level in total RNA in the kidney tissues. The mRNA **(B)** and protein **(C)** expression of ALKBH5 in renal tissue as revealed by RT-PCR and Western blot, respectively. **(D)** Quantifications of ALKBH5 protein expressions made by ImageJ software. Data are presented as mean ± SD (n = 6, **p < 0.01 vs. sham; #*p* < 0.05 vs. UUO group).

To investigate whether m6A modification occurred in UUO and if genistein involved in this process. We measured the total m6A level using UPLC-MS/MS analysis. The total m6A level increased after UUO; however, genistein pretreatment has a significant reduction methylation level (Figure 4A). In concurrence with this, we found that the mRNA levels of m6A demethylase ALKBH5 on the seventh day after UUO was significantly lower compared to the sham group, and increased in genistein pretreatment group (Figure 4B). The ALKBH5 protein expression results were consistent with the mRNA expression (Figures 4C,D).

### Snail Participates in m6A-Regulated Epithelial-to-Mesenchymal Transition in Renal Epithelial Cells

**FIGURE 5:**
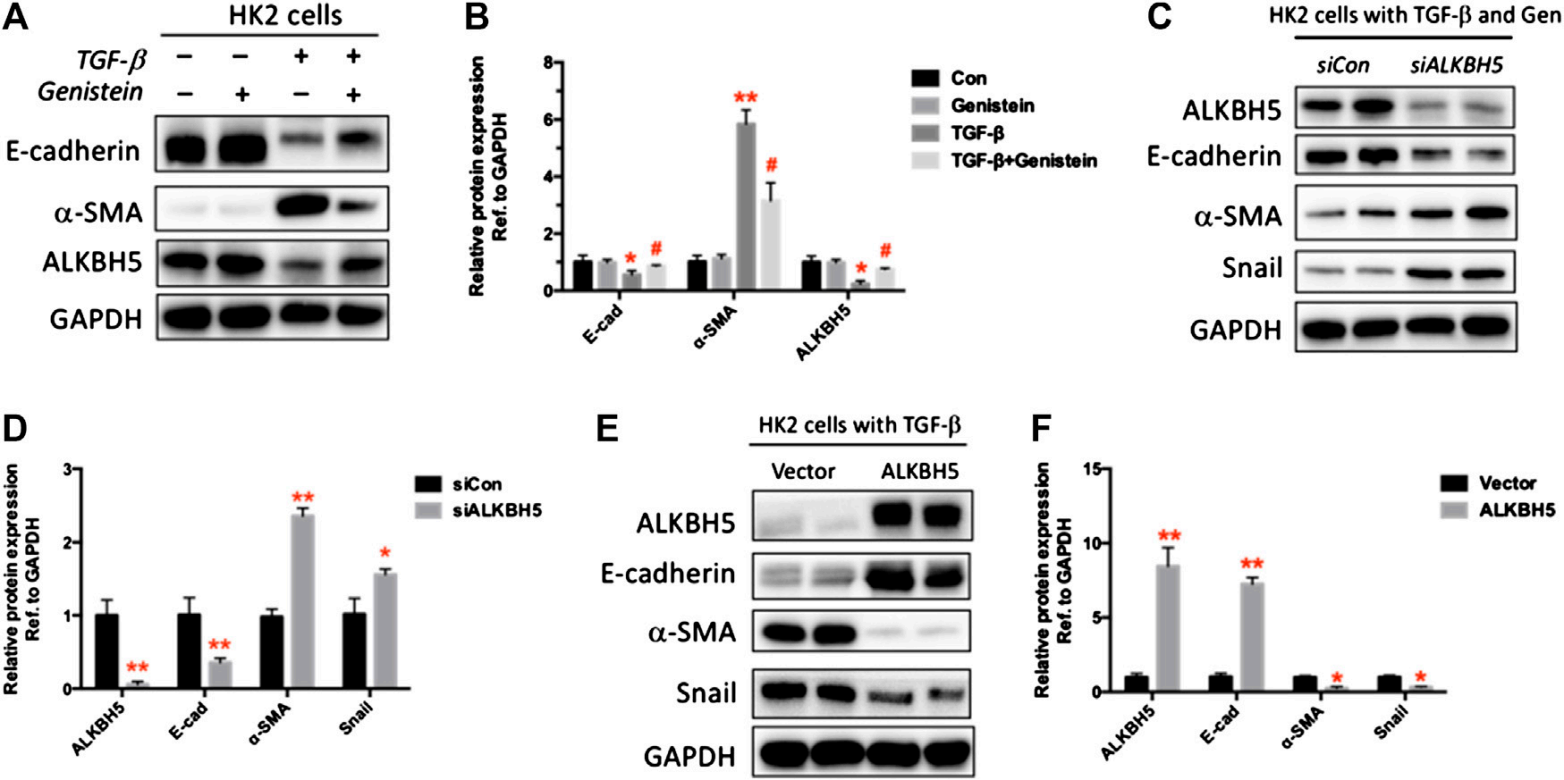
Snail is involved in m6A-regulated EMT in renal epithelial cells. **(A)** HK2 cells were treated with genistein (15 μM) with or without TGF-β (5 ng/ml) for 24 h. The protein expressions of E-cadherin, α-SMA, and ALKBH5 were assayed by Western blot. **(B)** Quantification of **(A)** (n = 3, *p < 0.05, **p < 0.01 vs. control; #*p* < 0.05 vs. TGF-β group). **(C)** HK2 cells were treated with genistein (15 μM) and TGF-β (5 ng/ml) with the synthesized human ALKBH5 siRNA oligonucleotides and the scrambled oligonucleotides (siCon) at final concentrations of 50 nM. The protein expressions of ALKBH5, E-cadherin, α-SMA, and snail as revealed by Western blot. **(D)** Quantification of **(C)** (n = 3, *p < 0.05, **p < 0.01 vs. siCon). **(E)** HK2 cells were transfected with pcDNA/ALKBH5 or vector control through liposome-mediated transfection. The protein expressions of ALKBH5, E-cadherin, α-SMA, and snail as revealed by Western blot. **(F)** Quantification of **(E)** (n = 3, *p < 0.05, **p < 0.01 vs. vector). Data are presented as mean ± SD.

Herein, we examined the function of genistein in the regulation of ALKBH5 and EMT marker proteins in renal tubule cells *in vitro*. We verify whether the anti-renal fibrosis impact of genistein was in relation to ALKBH5 restoration. The previous investigation reveals that TGF-β is critical in the development of renal fibrosis (Meng et al., 2016). We found that TGF-β treated renal tubule cells exhibited lower levels of ALKBH5, E-cadherin, and higher levels of α-SMA. However, these effects were reversed following genistein treatment (Figures 5A,B). These results were consistent with *in vivo* results. Further, to determine the underlying mechanisms of m6A-regulated EMT through changing the expression of ALKBH5 using silenced endogenous ALKBH5 and transfected with a plasmid expressing ALKBH5, we still measured EMT biomarkers and snail, a key transcription factor of EMT. Following siALKBH5 transfection, the expression of ALKBH5 was decreased; while an increase in expression was observed after transfection with the ALKBH5 expression vector (Figures 5C–F). We found that knockdown of ALKBH5 suppressed the E-cadherin expression and promoted the level of a-SMA and Snail, whereas overexpression of ALKBH5 showed opposite results (Figures 5C–F) Genistein could restore the expression of ALKBH5. If the ALKBH5 knockdown the anti-EMT effect of genistein has weakened and snail was increased (Figures 5C,D).

**FIGURE 6:**
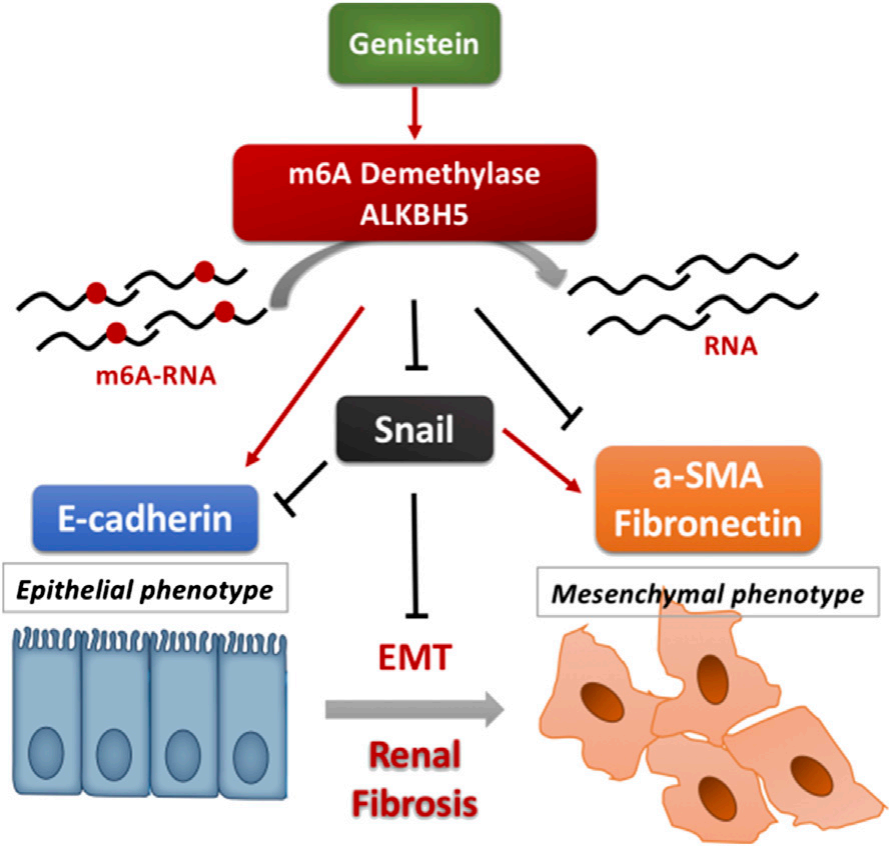
Graphical illustration of the mechanisms in the present study.

## Discussion

CKD is a significant cause of death (Webster et al., 2017). Renal tubule-interstitial fibrosis is a common pathway leading to ECM accumulation, organ scarring, and the progression of CKD (Humphreys, 2018). However, no effective therapy has not been found to counter these destructive disorders. An enhanced EMT characterized by the deposition of excessive amounts of ECM tends to occur particularly in severe tubular atrophy and tubule-interstitial fibrosis in the UUO mice (Bascands and Schanstra, 2005). Currently, there still a lack of effective treatments for renal fibrosis. Therefore, determining the mechanisms by which epithelial phenotype differentiate into the mesenchymal phenotype is critical to understanding how to prevent renal fibrosis.

Genistein is one of the main active factors in soybean isoflavones. It is the most effective functional component in soy isoflavone and has a variety of physiological functions. Notably, its biochemical properties have been tested extensively in the treatment of many disorders, including inflammation, cancer, and apoptosis (Mukund et al., 2017). Genistein plays a protective function in I/R-induced and cisplatin-induced renal injury (Sung et al., 2008; Li et al., 2017). As a natural plant extract, genistein is relatively safe as a bioactive supplement for the prevention and therapy of CKD. In this study, we found genistein involved UUO-induced EMT process and had anti-renal fibrosis actions, which is consistent with separation another observation by Li et al. (2019). We explore here whether other mechanisms be involved. Our results showed the effectiveness of genistein on the suppression of renal fibrosis. In this study, the physiological lesions and interstitial fibrosis were significantly recovered seven days after UUO following treatment with genistein. Moreover, the levels of ECM proteins, fibrogenic, and inflammatory factors (TGF-β, IL-1β, MCP-1, and TNF-α), as well as α-SMA and pSmad (EMT markers) were considerably lower in the genistein-treated mice compared to the UUO-only mice. The administration of genistein partially ameliorated inflammatory factors. These findings provide strong evidence that genistein could be effective in the treatment of obstructed kidney. Also, our results revealed that the protective effects of genistein in renal injury induced by UUO were related to a higher ALKBH5 expression, which is the m6A modification gene to remove the methylation. It offers novel insights into the treatment and prevention of UUO-induced renal interstitial fibrosis.

N6-Methyladenosine is the most robust modification of mammal mRNA (Cao et al., 2016). More and more literature reported the critical roles of m6A in epigenetic regulation and how this modification affected the pathogenesis of various diseases, including kidney injury. m6A was reported to be involved in the EMT of cancer cells and modulated via the methyltransferase METTL3, demethylase ALKBH5, and its reader YTHDF1 (Lin et al., 2019). In this study, we speculated that there was a change in m6A modification in the course of UUO-induced renal damage. We examined that genistein can cause epigenetic changes and regulate m6A related gene expression. The findings revealed that m6A exhibited an upregulation trend following UUO, and downregulation trends follow genistein treatment.

Furthermore, we assessed the demethylase and m6A methyltransferase levels involved in the m6A modification. Based on the results, m6A demethylase ALKBH5 was up-regulated in the genistein pretreatment of the UUO group. *In vitro* study, results were consistent with *in vivo* experiments. Thus, we believe that ALKBH5 is a crucial regulatory gene involved in how genistein protects renal fibrosis. The present research about ALKBH5-mediated m6A demethylation mainly focuses on the pathogenesis of multiple tumors, including cancers of the pancreas, ovary, lung, and breast (Wu et al., 2019; Zhu et al., 2019; Jin et al., 2020; Tang et al., 2020). A recent study showed that ALKBH5 expression was suppressed in renal carcinoma compared to adjacent healthy tissues. Low levels of ALKBH5 and FTO mRNA were associated with reduced overall as well as cancer-specific survival after nephrectomy (Strick et al., 2020). In this study, we found no remarkable difference between the UUO and the sham group regarding the levels of FTO. Recently, two reports found m6A methylation involved in acute kidney injury. METTL3, an essential m6A methyltransferase, was involved in keap1/Nrf2 pathway modulation in colistin-induced kidney injury via and attenuating oxidative stress and apoptosis (Wang et al., 2019). Another m6A methyltransferase METTL14 was reported to modulate the pathogenesis of acute renal ischemia/reperfusion-induced injury via suppressing YAP1 (Xu et al., 2020). However, because their target genes, and their operation cellular environments and pathological conditions are different, METTL3 and METTL14, the orthologous genes and same methylases may also be incomparable. The upregulation of METTL3 plays a protective function against apoptosis and oxidative stress that is induced by colistin. On the contrary, knockdown of METTL14 is likely to protect the kidney against IR-injury. More details need to confirm the close relationships between mRNA methylation and kidney injury, especially in CKD. In this study, we first examined whether and how ALKBH5 modulates renal fibrosis. Based on the previous research, we detected vital transcription factors associated with EMT, including slug, snail, twist, and zeb1, and found that snail was regulated by ALKBH5.

## Conclusions

In conclusion, our findings show that m6A participates in UUO-induced renal fibrosis. ALKBH5 is a crucial regulator for the m6A modification and protection of genistein for renal fibrosis. Genistein ameliorates renal fibrosis by restoring ALKBH5 to regulate EMT. The results of the present study may provide new insights into the function of m6A modification in CKD, which can assist in the development of new drugs for alleviating renal fibrosis. However, further studies should be conducted to ascertain whether genistein can be utilized for the treatment and prevention of CKD.

## Data Availability Statement

The raw data supporting the conclusions of this article will be made available by the authors, without undue reservation.

## Ethics Statement

The animal study was reviewed and approved by Animal Care and Use Committee of Fudan University.

## Author Contributions

YN conceived and designed the experiments. YN and YS performed the experiments. YN and JC analyzed the data. NS, YF, and XY contributed reagents/materials/analysis tools. YN and XD wrote the manuscript.

## Funding

This study was supported by research grants 81501194 and 91849123 from National Natural Science Foundation of China and the Science and Technology Commission of Shanghai Municipality (14DZ2260200, the project of Shanghai Key Laboratory of Kidney and Blood Purification). The funders had no roles in study design, data collection and analysis, decision to publish, or preparation of the manuscript.

## Conflict of Interest

The authors declare that the research was conducted in the absence of any commercial or financial relationships that could be construed as a potential conflict of interest.
