# Supplementary figures and images for: Genistein Ameliorates Renal Fibrosis Through Regulation Snail via m6A RNA Demethylase ALKBH5
Source: Front Pharmacol. 2020 Nov 19;11:579265. doi: 10.3389/fphar.2020.579265 (PMC7751752; doi:10.3389/fphar.2020.579265)

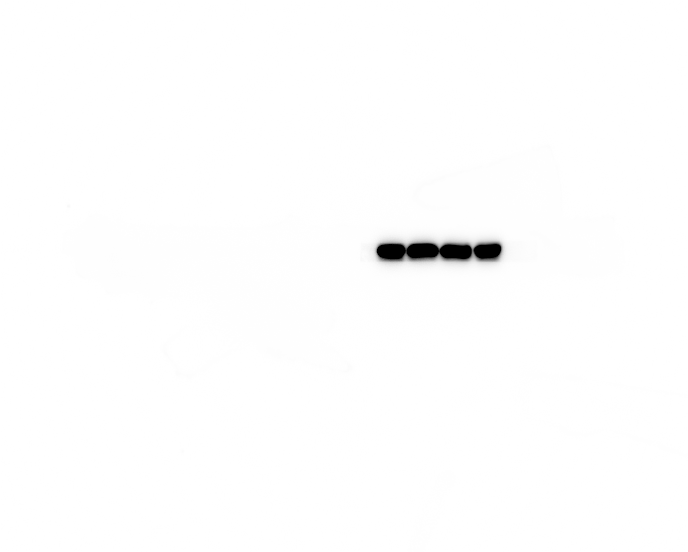

Supplement: Supplementary file 1 [file data_sheet1.zip › Images of the original western blots/Fig5E GAPDH.tif]

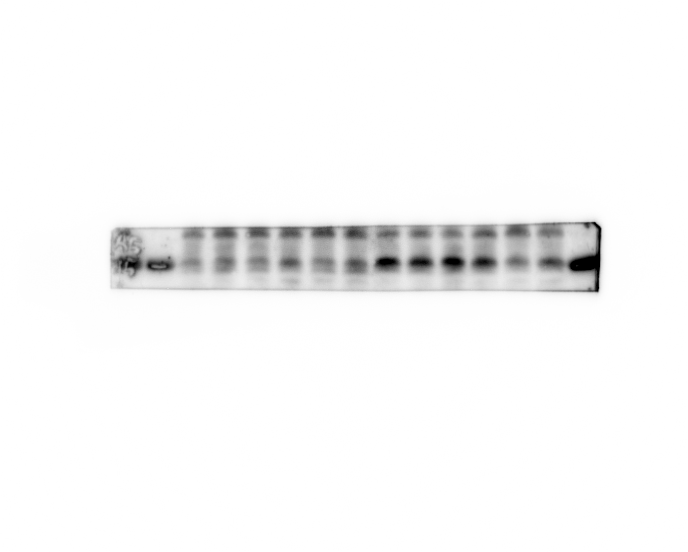

Supplement: Supplementary file 1 [file data_sheet1.zip › Images of the original western blots/Fig3A TGF-beta.tif]

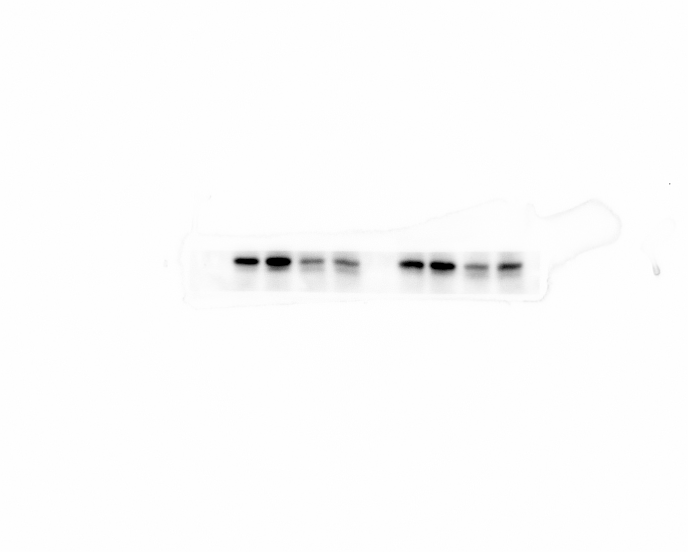

Supplement: Supplementary file 1 [file data_sheet1.zip › Images of the original western blots/Fig5C ALKBH5.tif]

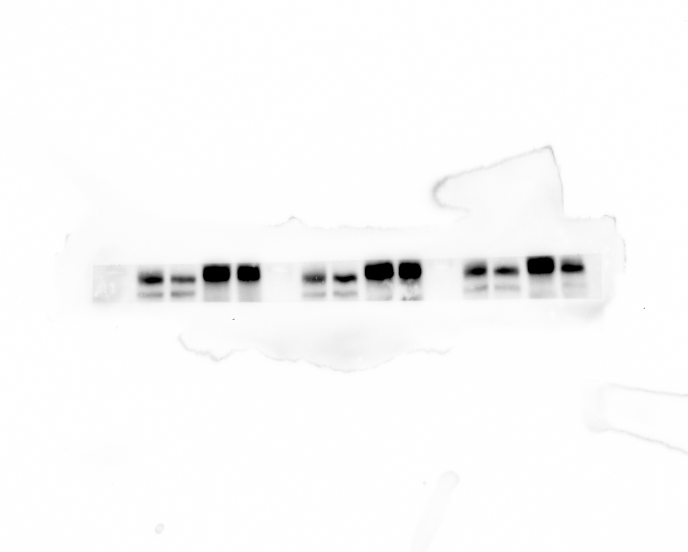

Supplement: Supplementary file 1 [file data_sheet1.zip › Images of the original western blots/Fig5A E-cad.tif]

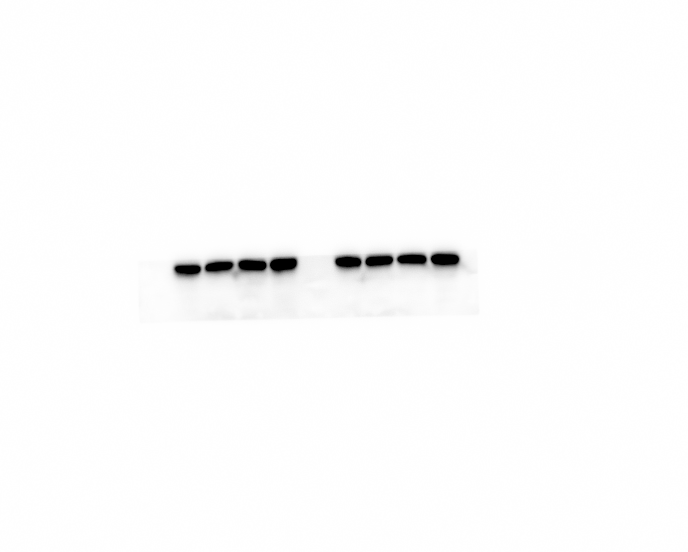

Supplement: Supplementary file 1 [file data_sheet1.zip › Images of the original western blots/Fig5C GAPDH.tif]

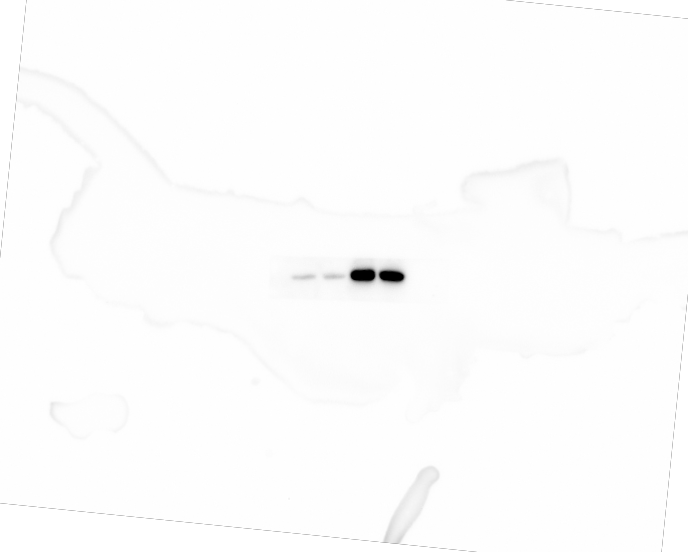

Supplement: Supplementary file 1 [file data_sheet1.zip › Images of the original western blots/Fig5E a-SMA.tiff]

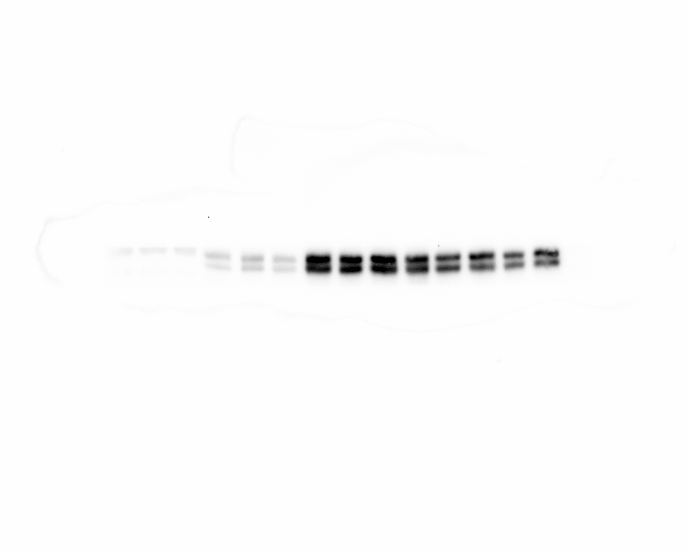

Supplement: Supplementary file 1 [file data_sheet1.zip › Images of the original western blots/Fig3A pSmad.tif]

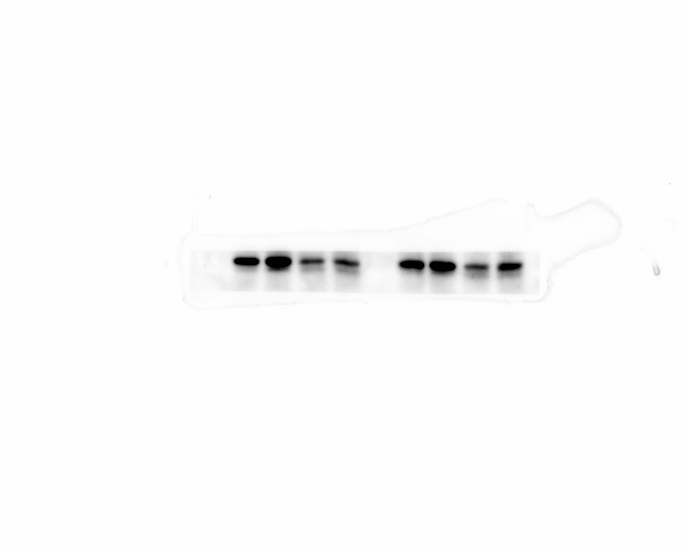

Supplement: Supplementary file 1 [file data_sheet1.zip › Images of the original western blots/Fig5A ALKBH5.tif]

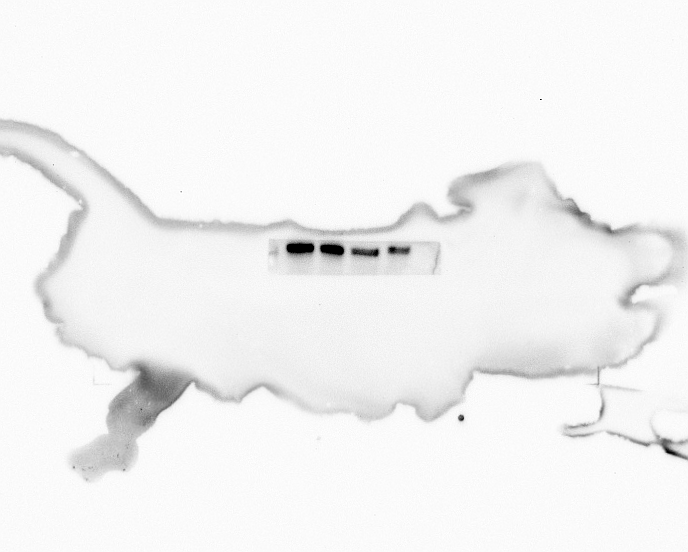

Supplement: Supplementary file 1 [file data_sheet1.zip › Images of the original western blots/Fig5E Snail.tif]

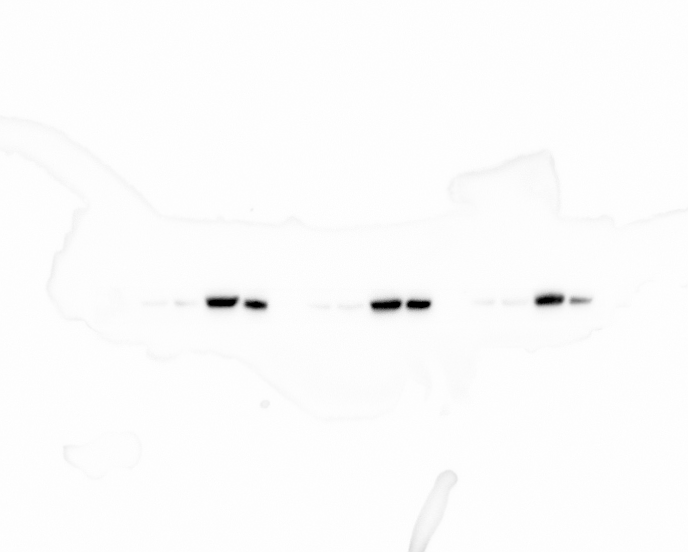

Supplement: Supplementary file 1 [file data_sheet1.zip › Images of the original western blots/Fig5A a-Sma.tif]

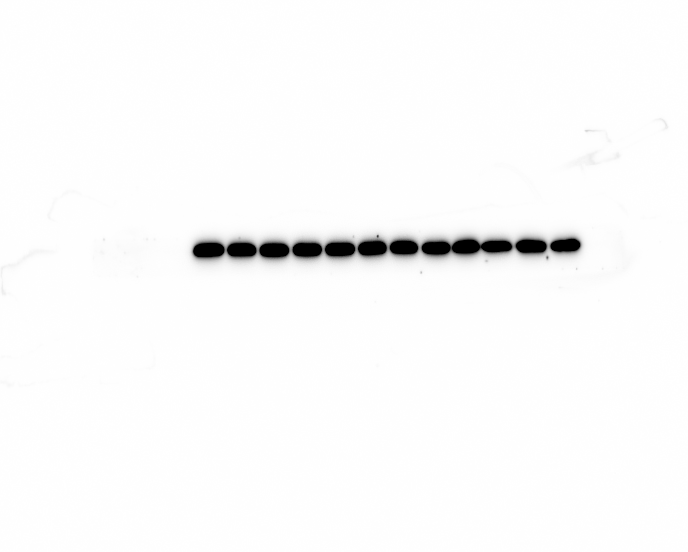

Supplement: Supplementary file 1 [file data_sheet1.zip › Images of the original western blots/Fig3A GAPDH.tif]

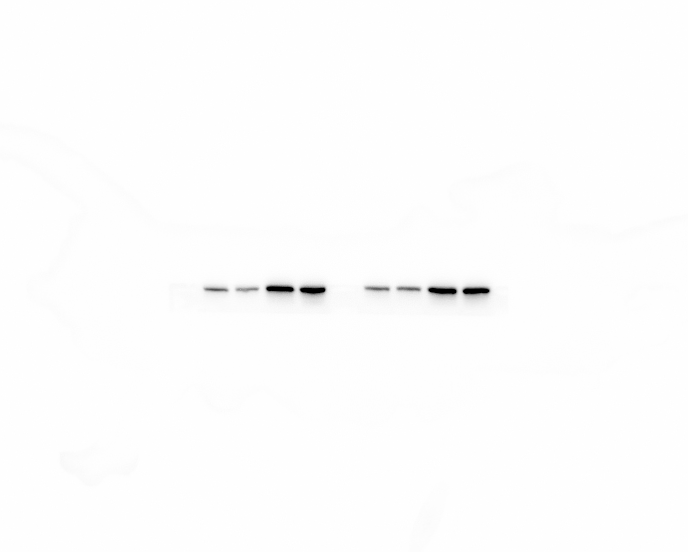

Supplement: Supplementary file 1 [file data_sheet1.zip › Images of the original western blots/Fig5C Snail.tif]

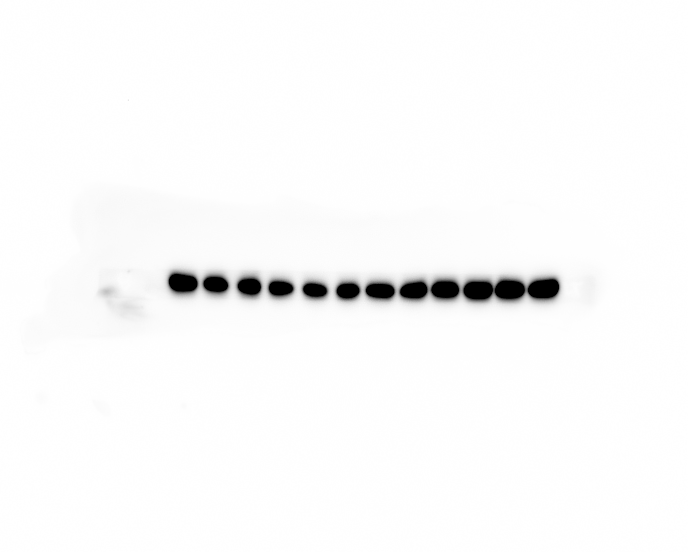

Supplement: Supplementary file 1 [file data_sheet1.zip › Images of the original western blots/Fig4C GAPDH.tif]

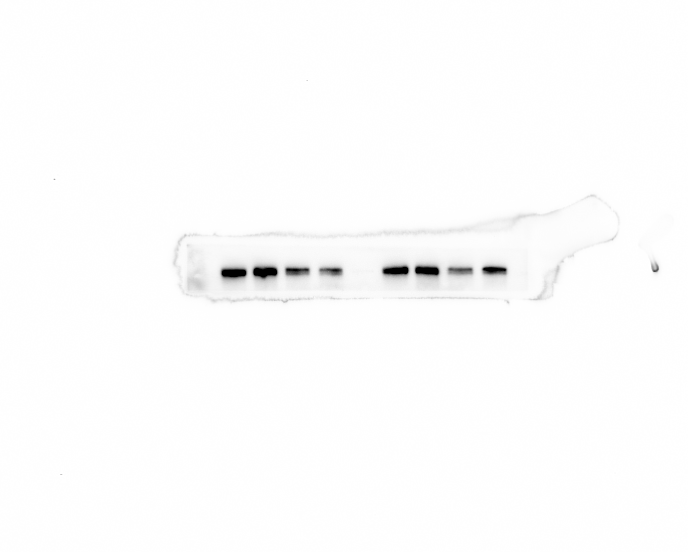

Supplement: Supplementary file 1 [file data_sheet1.zip › Images of the original western blots/Fig5C E-cad.tif]

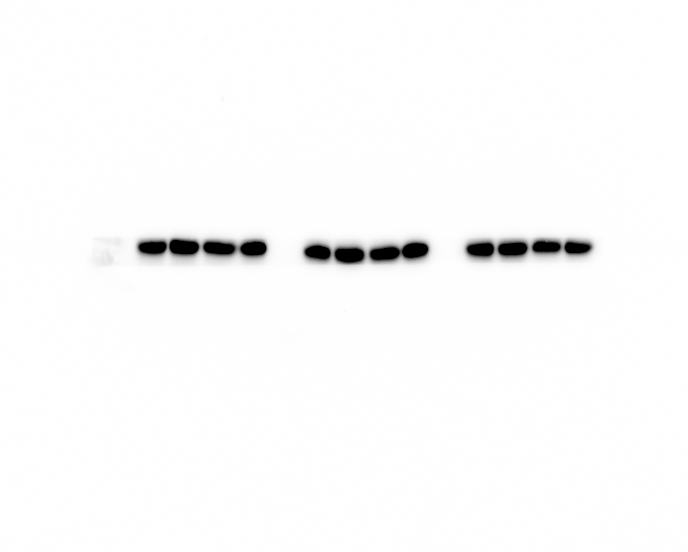

Supplement: Supplementary file 1 [file data_sheet1.zip › Images of the original western blots/Fig5A GAPDH.tif]

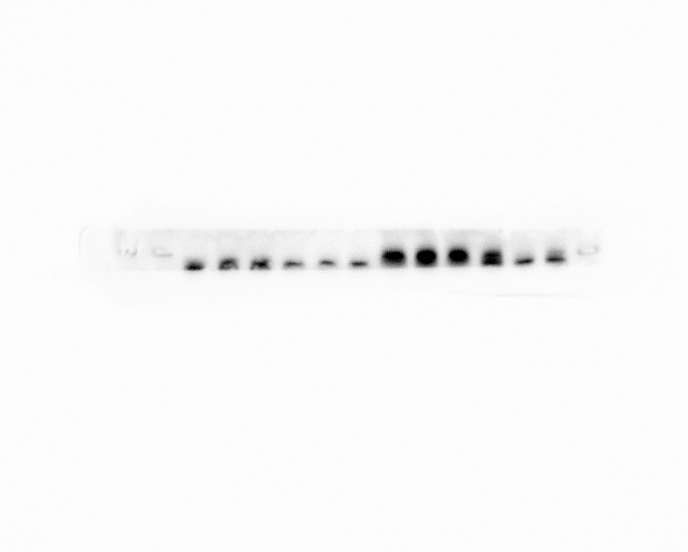

Supplement: Supplementary file 1 [file data_sheet1.zip › Images of the original western blots/Fig3A a-SMA.tif]

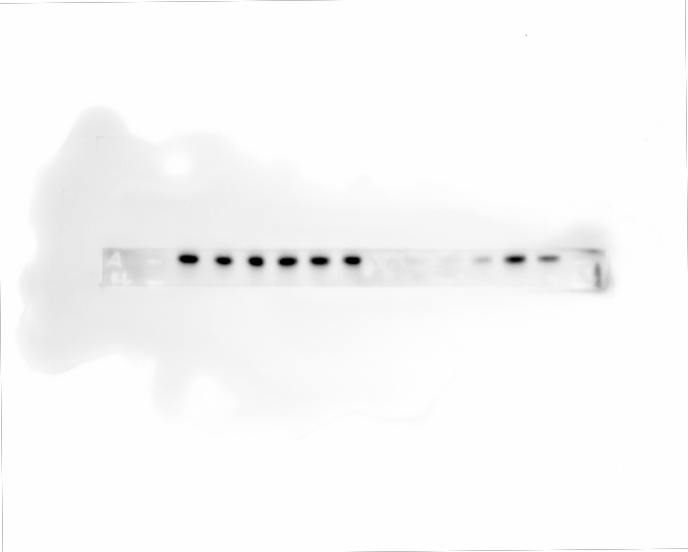

Supplement: Supplementary file 1 [file data_sheet1.zip › Images of the original western blots/Fig4C ALKBH5.tiff]

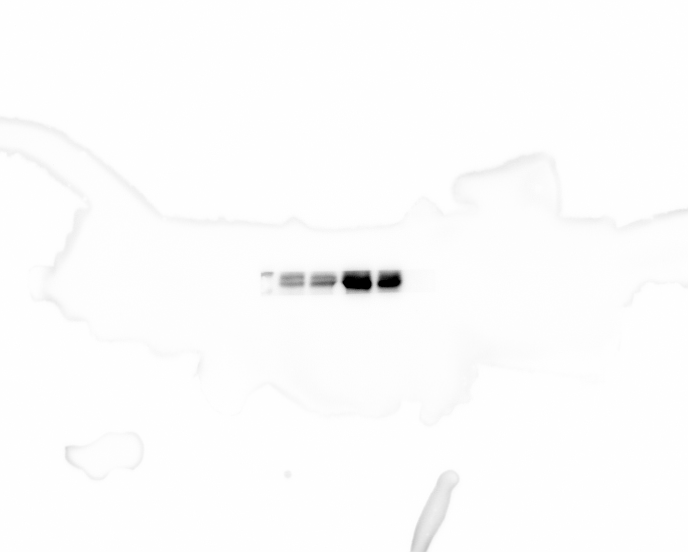

Supplement: Supplementary file 1 [file data_sheet1.zip › Images of the original western blots/Fig5E E-cad.tif]

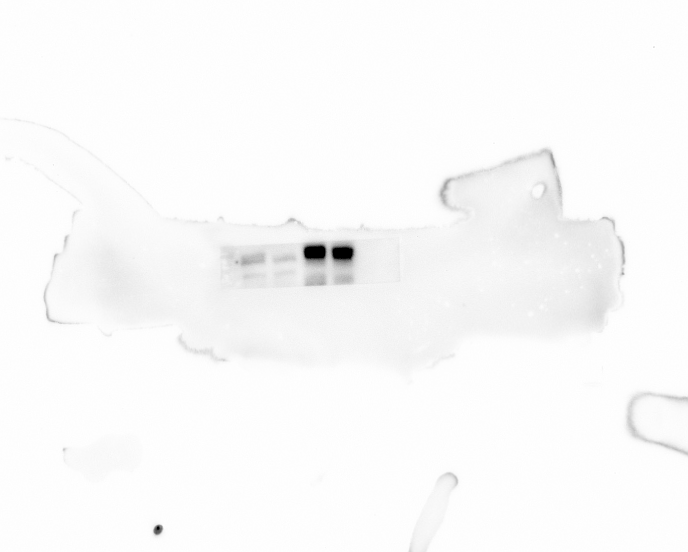

Supplement: Supplementary file 1 [file data_sheet1.zip › Images of the original western blots/Fig5E ALKBH5.tif]

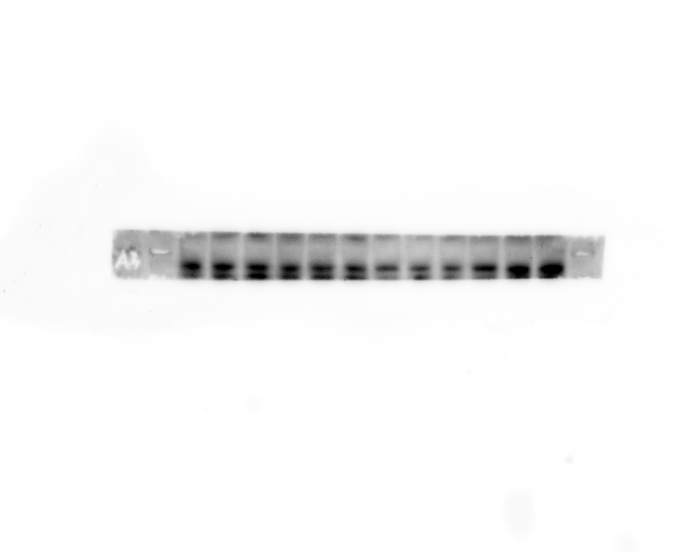

Supplement: Supplementary file 1 [file data_sheet1.zip › Images of the original western blots/Fig3A E-cad.tif]

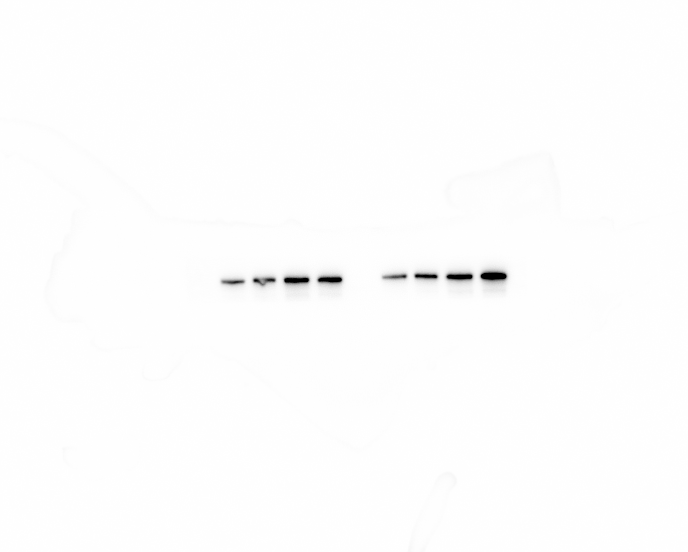

Supplement: Supplementary file 1 [file data_sheet1.zip › Images of the original western blots/Fig5C a-SMA.tif]
